# MiR-20a promotes osteogenic differentiation in bone marrow-derived mesenchymal stem/stromal cells and bone repair of the maxillary sinus defect model in rabbits

**DOI:** 10.3389/fbioe.2023.1127908

**Published:** 2023-04-05

**Authors:** Yi-Xuan Wang, Zhu-Li Peng, Zhi-Wen Sun, Yan-Jun Pan, Hong Ai, Zhi-Hui Mai

**Affiliations:** ^1^ Department of Stomatology, The Third Affiliated Hospital of Sun Yat-Sen University, Guangzhou, China; ^2^ Department of Orthodontics, Affiliated Stomatology Hospital of Guangzhou Medical University, Guangzhou, China

**Keywords:** miR-20a, osteogenesis, bone marrow-derived mesenchymal stem/stromal cells (BMSCs), bone morphogenetic protein 2 (BMP2), bone repair

## Abstract

**Introduction:** This study aimed to determine whether miR-20 promoted osteogenic differentiation in bone marrow-derived mesenchymal stem/stromal cells (BMSCs) and accelerated bone formation in the maxillary sinus bone defect model in rabbits.

**Methods:** BMSCs were transfected with miR-20a or anti-miR-20a for 12 h, followed by detection of RUNX2, Sp7 mRNA, bone morphogenetic protein 2 (BMP2), and RUNX2 protein expression. Alkaline phosphatase (ALP) activity and Alizarin Red S staining were used to detect calcified nodule deposition. In the rabbit maxillary sinus bone defect model, miR-20a loaded with AAV and BMP2 protein were mixed with Bio-Oss bone powder for filling the bone defect. At 4 weeks and 8 weeks, bone density was detected by cone beam computed tomography (CBCT), and new bone, osteoblasts, and collagen type 1 were evaluated by hematoxylin and eosin (HE) staining and immunohistochemical (IHC) staining.

**Results:** Overexpression of miR-20a enhanced the mRNA and protein levels of BMP2, RUNX2, and SP7, the activity of ALP, and the levels of matrix mineralization, whereas the levels and activity of the aforementioned factors were decreased by anti-miR-20a treatment of BMSCs. Furthermore, miR-20a significantly increased the bone density, the number of osteoblasts, and the secretion of collagen type 1 in bone defects compared with Bio-Oss bone powder in the rabbit maxillary sinus bone defect model.

**Conclusion:** Overall, miR-20a can induce osteogenic differentiation in BMSCs and accelerate bone formation of maxillary sinus defects in rabbits.

## Introduction

Many patients who require an implant in the maxillary posterior tooth area suffer from an insufficient height of the residual alveolar bone, which may be due to maxillary sinus gasification, alveolar bone atrophy, and other reasons. Generally, maxillary sinus floor elevation is a common option to increase the height of the alveolar bone (Danesh-Sani et al., 2016; Bathla et al., 2018). Approximately 63% of patients have received maxillary sinus floor elevation combined with bone augmentation materials (Cha et al., 2016). Various bone augmentation and tissue engineering materials from biological or synthetic sources have been developed for oral implants to reduce or avoid complications and achieve better outcomes. A meta-analysis by Da Rosa et al. revealed that the application of bioactive protein and the platelet concentrate alone or in combination with maxillary sinus floor elevation did not affect bone density increase results but promoted the degradation of residual bone density increase materials, reduced the healing time, and relieved postoperative symptoms (Guo et al., 2020; Meng et al., 2020).

Bone morphogenetic protein 2 (BMP2) plays a significant role in the formation and development of bone and cartilage. Studies have revealed the osteogenic and osteoinductive characteristics of recombinant human BMP2 (rhBMP2) and provided support for the application of rhBMP2 as a substitute for autologous bone in maxillary sinus floor elevation (Lee et al., 2013; Mai et al., 2013). Moreover, compared with the simple autologous bone transplantation group, a higher final bone density could be observed when rhBMP2 was used as an autologous bone graft substitute; in addition, the combined application of BMP2 and transforming growth factor-1 α (TGF-1α) significantly increased the degree of maxillary sinus osteogenesis. However, with the application of BMP2, many side effect profiles have emerged clinically. First, the high dose of BMP2 induces an inflammatory reaction in the body; second, the time BMP2 remains in the body is too short to be supplemented in time due to the presentation mode of BMP2. To solve aforementioned limitations, BMP2 equipped with sustained-release agents has been studied widely. However, no accepted sustained-release agents have been found yet.

Related studies proved the involvement of microRNAs in the regulation of many different functions, and at present, thousands of microRNAs have been identified. The microRNAs involved in bone remodeling include miR-20a, miR-21, and miR-335-5p (Mai et al., 2013; Wang et al., 2018; Li et al., 2020; Xiao et al., 2021). A study demonstrated that miR-20a inhibits the proliferation and differentiation of osteoclasts by targeting RANKL *via* the TLR4/p38 pathway (Kong et al., 2021). Our previous studies demonstrated that fluid shear stress (FSS) induced osteogenic differentiation of bone marrow-derived mesenchymal stem/stromal cells (BMSCs). In addition, we found that multiple microRNAs, including miR-20a, were significantly differentially expressed during steady FSS-mediated osteogenic differentiation through high-throughput gene chip screening. Furthermore, miR-20a could enhance FSS-induced osteoblast differentiation by activating the BMP2 signaling pathway (Mai et al., 2022). Therefore, we attempted to determine if miR-20 could promote osteogenic differentiation of BMSCs through activating BMP2/Runx2 signaling.

## Materials and methods

### Cell culture

BMSCs were cultured in α-MEM media (Life Technologies, Grand Island, NY, United States) containing 10% fetal bovine serum (FBS; Life Technologies) and 1% penicillin-streptomycin (Life Technologies) and maintained in a 5% CO_2_ humidified environment at 37°C. The medium was replaced every 3 days. Osteogenic differentiation of BMSCs was induced with an osteogenic medium containing 50 μg/mL ascorbic acid, 10 mM β-glycerophosphate, and 0.1 µM dexamethasone.

### Oligonucleotide transfection

BMSCs were seeded in 24-well plates and transfected with miR-20a mimic (100 nM), miR-20a inhibitor (anti-miR-20a, 100 nM), and negative control (NC, 100 nM), using Lipofectamine RNAiMAX (Invitrogen), according to the manufacturer’s instructions. After transfection for 12 h, the culture medium was changed to an osteogenic medium. The efficiency of transfection was assessed using Cy3-labeled oligonucleotide RNA mimics. Cy-3 miRNA mimics labeled by red fluorescence were transfected into BMSCs to supervise the transfection rate at 12 h.

### RNA isolation, reverse transcription, and quantitative real-time PCR

Total RNA was extracted using TRIzol reagent (Life Technologies). Expression of mature miRNAs was determined by stem-loop primer SYBR Green quantitative real-time-PCR (qRT-PCR) and normalized to U6. Transcriptional levels of the tested genes were detected by qRT-PCR and normalized to GAPDH using a 7500 Real-Time PCR system (Applied Biosystems, United States) and Platinum SYBR Green qPCR SuperMix-UDG (Life Technologies). Next, the relative mRNA levels were measured using the comparative CT method (^△△^CT) with GAPDH as an internal control. In addition, the qRT-PCR protocol consisted of 40 cycles (94°C for 15 s, 60.5°C for 15 s, 72°C for 15 s) after an initial denaturation step (94°C for 2 min).

### Alkaline phosphatase (ALP) activity assay and staining

The transfected cells were lysed on days 6 and 9 with a lysis buffer composed of 20 mM Tris-HCl (pH 7.5), 150 mM NaCl, and 1% Triton X-100. Taking p-nitrophenyl phosphate as the substrate (LabAssay^TM^ ALP, alkaline phosphatase; Wako, Japan), cellular ALP activity was determined using the p-nitrophenyl phosphate and disodium salt (PNPP) method, according to the manufacturer’s instructions. After excluding the background level, the enzyme activity (units/mg protein) was equal to the concentration of p-nitrophenol (nmol/mL) released by the sample within 15 min. The ALP activity of each sample was normalized to the protein concentration measured using a BCA protein assay kit (Thermo Scientific Pierce). Next, the transfected cells were washed and fixed with 4% paraformaldehyde on day 9; then, 3% ALP staining solution composed of nitrobule tetrazolium (NBT) and 5-bromo-4-chloro-3-indolephosphate (BCIP) (Gibco, United States) was used for staining. Subsequently, five pictures were randomly selected from each section under a light microscope (400 × Olympus, Japan). Finally, the relative integrated optical density (IOD) of ALP staining was calculated by Image-Pro Plus 6.0 software.

### Alizarin Red S staining

To detect extracellular matrix (ECM) mineralization as a marker of terminal differentiation, cells transduced with miR-20a or anti-miR-20a were seeded into 6-well tissue culture plates and induced with an osteogenic medium. On day 14, cells were washed and then fixed with 4% paraformaldehyde and stained with 1% Alizarin Red S solution (Sigma-Aldrich). Next, images were randomly taken using a light microscope (400 ×, Olympus), quantification of Alizarin Red S staining was performed *via* extraction with cetylpyridinium chloride monohydrate (TCI, Japan), and absorbance was measured at 560 nm.

### Western blotting

After quantification using a BCA protein assay kit (Thermo Scientific Pierce, United States), cell lysates were separated by SDS-PAGE (12%) and transferred to PVDF membranes. The membranes were blocked with 5% non-fat dry milk for 1 h and then incubated with primary antibodies against BMP2 (1:400; Bioss Company, China) or RUNX2 (1:400; Bioss Company) overnight. Subsequently, the membranes were washed and incubated with a horse radish peroxidase (HRP)-conjugated secondary antibody (Santa Cruz Biotechnology), followed by detection through chemiluminescence. GAPDH protein was used as an internal loading control. The intensity of the protein fragments was quantified by Image Lab software (version 4.0, Bio-Rad, United States).

### Experimental animals

A total of 12 male New Zealand rabbits (age: 20 ± 2 weeks, weight: 2.0 ± 0.5 kg) were purchased from the Longgui Xingke Animal Farm, Baiyun District, Guangzhou, China. They were housed in a single cage with constant temperature and humidity and had free access to water. All animal experiments were conducted in accordance with the standards of the Laboratory Animal Guide.

### Study design

After routine anesthesia and skin preparation, a 1-cm incision was made above the nasal ridge and the periosteum was reflected to expose the outer wall of the maxillary sinus on both sides. The bone plate of the lateral maxillary sinus walls was removed with a dental ball drill, resulting in a bone defect with a diameter of 1 cm. For avoiding perforation, the maxillary sinus mucosa was completely preserved during surgery. After removing the lateral wall, the periosteum was observed to float up and down evenly with the breathing of the rabbits.

The 12 New Zealand rabbits (24 maxillary sinuses) were randomly divided into four groups (three rabbits and six maxillary sinuses per group). A small amount of rabbit blood was mixed with the prepared bone powder. To be specific, the Bio-Oss group (negative control) rabbits were given 0.15 g Bio-Oss bone powder; the miR-20a group rabbits were treated *via* 0.15 g Bio-Oss bone powder +40 µL (titer 6 × 1,011 vector genome (vg)/mL) adeno-associated virus (AAV) carrying miR-20a; the BMP2 group (positive control) rabbits were administered with 0.15 g Bio-Oss bone powder +25 µL rhBMP2; and the BMP2+miR-20a group rabbits were given 0.15 g Bio-Oss bone powder +40 µL (titer 6 × 1,011 (vg)/mL) AAV carrying miR-20a and 25 µL rhBMP2. The Bio-Oss bone powder was obtained from Bio-Oss, Geistlich, Switzerland; AAV carrying miR-20a was obtained from Shanghai Jikai Gene, China; and BMP2 was obtained from Tonbo Biosciences, United States.

**FIGURE 1:**
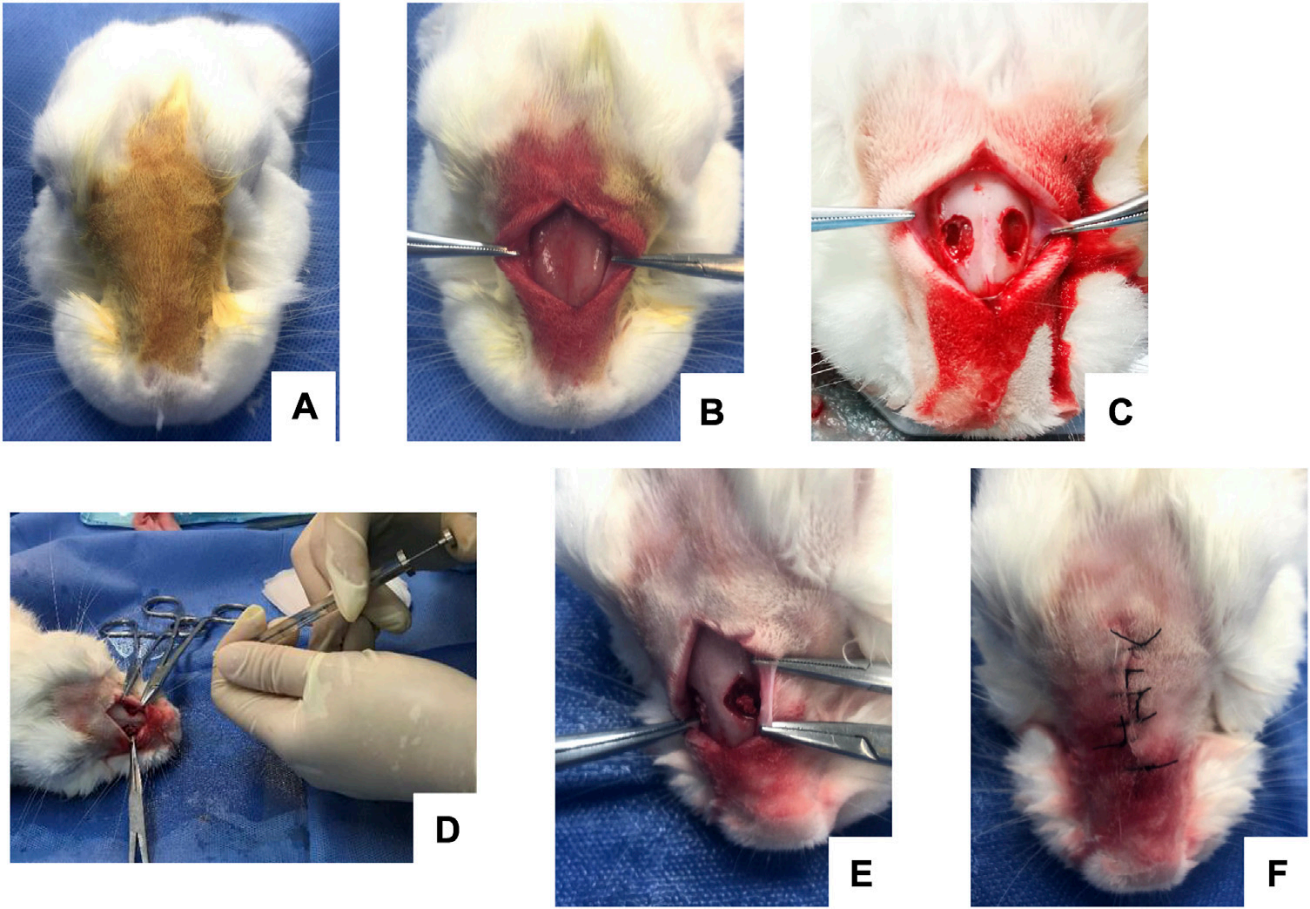
Intraoperative images of maxillary sinus elevation in rabbits. **(A)** Routine anesthesia and local disinfection. **(B)** Periosteum stripping to fully expose the nasal bone and nasal incisor suture. **(C)** Creation of a round bone window on both sides of the nasal suture to expose the maxillary sinus membrane. **(D)** Injection with a microsyringe. **(E)** Placement of the blood/bone powder mixture. **(F)** Suture of the operative incision.

In each group, the blood/bone powder mixtures were carefully placed in the bone defect areas. Attention was paid to the breathing rhythm of the rabbit to prevent the mixture from slipping when placing the mixture into the bone defect. The Bio-Gide periosteum was covered, and the surgical area was disinfected after incision sutures to prevent wound infection. Finally, the rabbits were continuously injected with gentamicin intramuscularly for 4 days. Images of the operative procedures are shown in Figures 1A–F.

### Cone beam spiral computed tomography (CBCT)

At weeks 4 and 8 after surgery, experimental rabbits were killed for gross observation and measurement of the elevation height of the maxillary sinus by cone beam computed tomography (CBCT). The skull and maxillofacial bone of the rabbits were scanned by CBCT at the Department of Stomatology, the Third Affiliated Hospital of Sun Yat-sen University. Thereafter, the gray values of the bottom, middle, and surface of the maxillary sinus operating area were measured by the same physician using NNT viewer software.

### Hematoxylin and eosin (H&E) staining and immunohistochemical (IHC) staining

Bone tissue in the operating area of the maxillary sinus was collected for microscopic examination after hematoxylin and eosin (H&E) staining and immunohistochemical (IHC) staining. For HE staining, bone tissue from the maxillary sinus lift area was fixed, dehydrated, decalcified, embedded, sectioned, stained, and then observed under a microscope. For IHC evaluation, bone tissue of the maxillary sinus lift surgery area was dehydrated and decalcified. After dewaxing, antigen repair was carried out according to the concentration of Collagen Ⅰ Monoclonal Antibody (COL-Ⅰ; Invitrogen, United States) instructions. Finally, IHC staining was performed on the treated tissues.

### Statistical analysis

Data were presented as the mean ± standard deviation (SD) of the three experiments. A two-tailed *t*-test or one-way ANOVA was used for comparisons of experiments with more than two subgroups. *p* < 0.05 was indicated statistically significant.

## Results

### Overexpression of miR-20a promotes the differentiation of bone marrow-derived mesenchymal stem/stromal cells (BMSCs)

**FIGURE 2:**
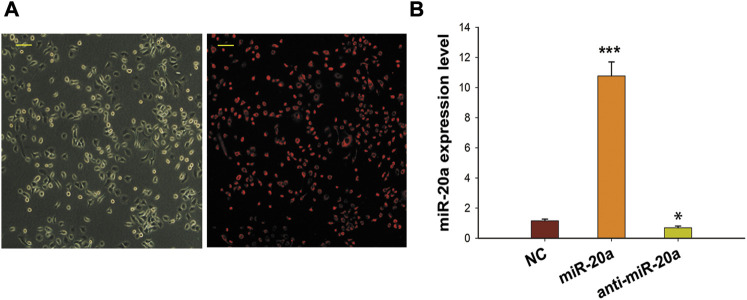
Effective transfection of synthetic miRNA in BMSCs. **(A)** Gray scale phase contrast images (before transfection) and the red visual fields (after transfection) showed cells with Cy-3 fluorescence. Cy-3 miRNA mimics labeled by red fluorescence were transfected into BMSCs to supervise the transfection rate at 12 h. The transfection efficiency (90%) was determined as *E* = Cy-3-positive cell number/total cell number. Magnification ×400. Scale bar = 50 µm. **(B)** qRT-PCR was applied to detect the miR-20a expression levels after miR-20a mimic or anti-miR-20a transfection. *p < 0.05 vs. NC. ***p < 0.001 vs. NC.

To clarify the biological function of miR-20a in osteogenic differentiation, overexpression and inhibition trials were conducted in this study. Transfection efficiency was determined as *E* = Cy-3-positive cell number/cell number in phase contrast. As shown in Figure 2A, the transfection rate was >90%. In addition, qRT-PCR results showed that the intracellular miR-20a content in BMSCs transfected with miR-20a mimic was about 10-fold higher than that in cells transfected with NC, while the level of miR-20a expression in the anti-miR-20a group was lower than that in the NC group (Figure 2B). These results indicated that miR-20a was successfully over or underexpressed in BMSCs.

### miR-20a stimulates osteogenic differentiation of bone marrow-derived mesenchymal stem/stromal cells (BMSCs)

**FIGURE 3:**
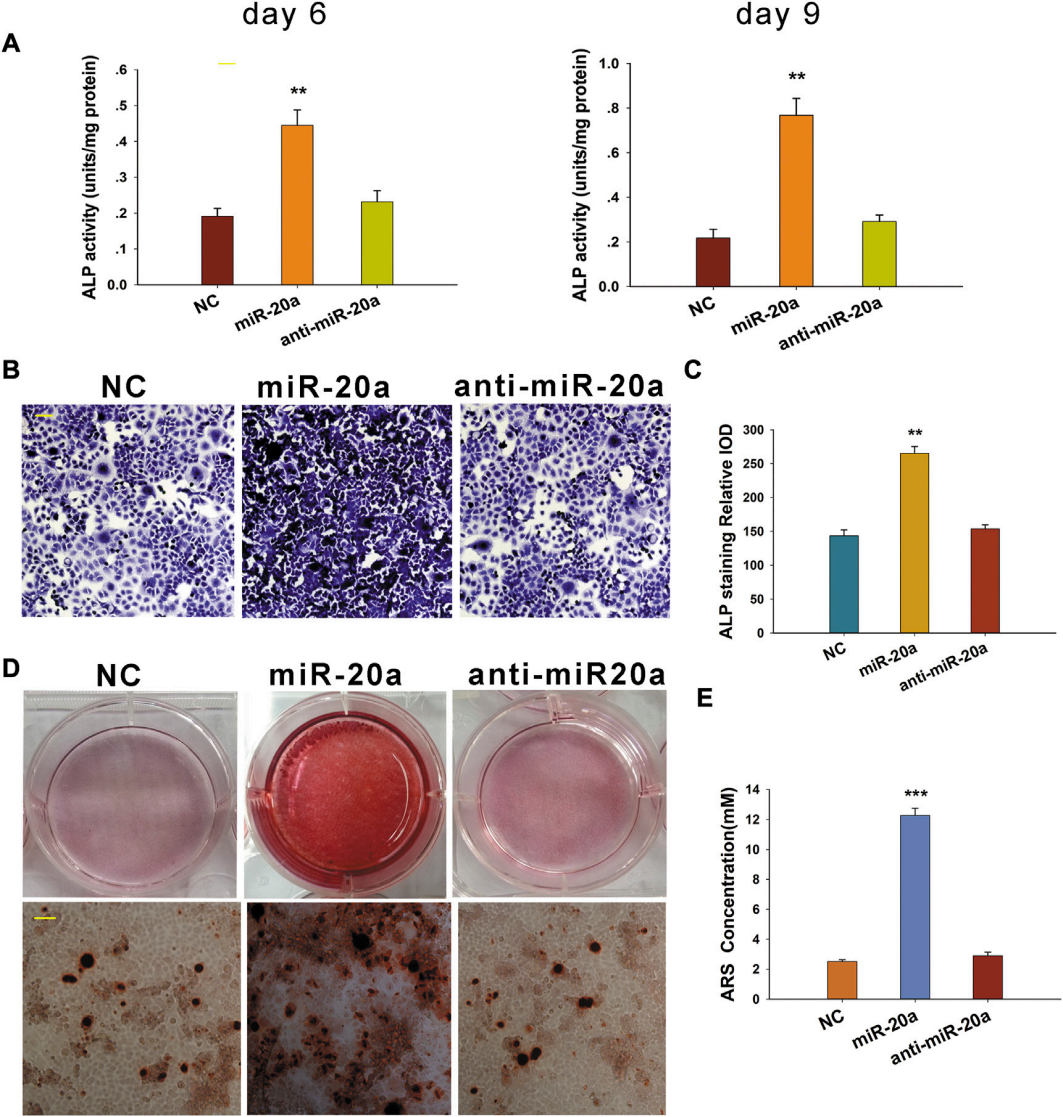
miR-20a promotes osteogenic differentiation of bone marrow-derived mesenchymal stem/stromal cells (BMSCs). **(A)** Alkaline phosphatase (ALP) activity was notably upregulated in cells with miR-20a overexpression on days 6 and 9. **(B)** ALP staining in miR-20a overexpressed cells became deeper on day 9. **(C)** Quantification of ALP staining was calculated. **(D)** Dyeing depth of Alizarin Red S staining in miR-20a overexpressed cells was darker on day 14. **(E)** Quantification of Alizarin Red S (ARS) staining was determined. Magnification ×400. Scale bar = 50 μm **p < 0.01 vs. NC. ***p < 0.001 vs. NC.

After transfection, BMSCs were cultured in a medium supplemented with osteogenic medium. ALP activity was detected at days 6 and 9. To be specific, miR-20a could obviously enhance ALP activity, whereas the ALP activity was not significantly different between the anti-miR-20a and NC groups (Figure 3A). Similar results of ALP activity were observed by ALP staining on day 9 (Figures 3B,C). To further validate the osteogenic effect of miR-20a, Alizarin Red S staining was performed on cells. Matrix mineralization was enhanced after overexpression of miR-20a on day 14, whereas the level of matrix mineralization was not significantly different between the anti-miR-20a and NC groups (Figures 3D,E). In a nutshell, miR-20a stimulated BMSC osteogenic differentiation.

### miR-20a activates the BMP2/RUNX2 signaling pathway

**FIGURE 4:**
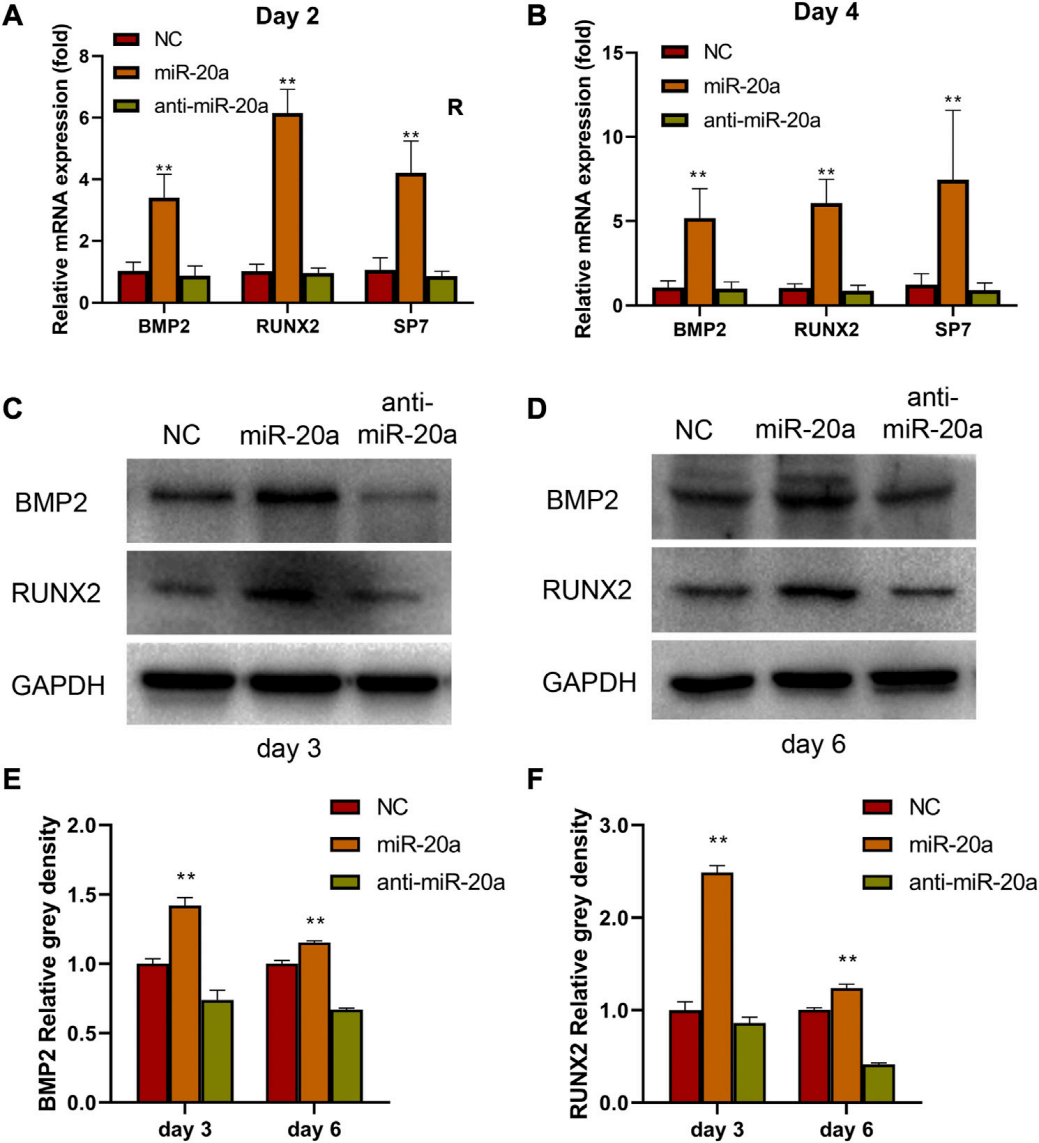
miR-20a activates the BMP2/RUNX2 signaling pathway. Bone marrow-derived mesenchymal stem/stromal cells (BMSCs) were transfected with 100 nM NC, miR-20a mimic, or anti-miR-20a. After transfection, osteogenesis was induced by cell culture in osteogenic medium. The mRNA levels of bone morphogenetic protein 2 (BMP2), RUNX2, and SP7 in miR-20a overexpressed cells were raised on day 2 **(A)** and day 4 **(B)**. Protein levels of BMP2 **(C and E)** and RUNX2 **(D and F)** were increased sharply in overexpressed miR-20a cells on days 3 and 6. *p < 0.05 vs. NC. **p < 0.01 vs. NC.

In this study, the function of miR-20a was detected by investigating the osteogenic effects of the loss or overexpression of miR-20a on BMSC differentiation *in vitro.* As shown in Figures 4A,B, overexpression of miR-20a improved the mRNA levels of *BMP2, RUNX2*, and *SP7* on days 2 and 4*.* In contrast, anti-miR-20a suppressed the mRNA levels of *BMP2, RUNX2*, and *SP7* on days 2 and 4. In addition, the BMP2 and RUNX2 protein levels were also detected on days 3 and 6. In comparison with the NC group, the BMP2 and RUNX2 protein levels were significantly increased in the miR-20a group, while they were notably decreased in the anti-miR-20a group (Figures 4C–F). Collectively, upregulation of miR-20a promotes osteogenic medium-induced osteogenic differentiation *via* the BMP2/RUNX2 signaling pathway.

### Postoperative condition of rabbits

Maxillary sinus lift surgery was successfully carried out on all rabbits. The food intake, activity, and weight of all rabbits returned to normal by 2 weeks after the operation. In addition, all rabbits survived the predetermined time. Purulent inflammation occurred in each maxillary sinus region in the Bio-Oss, miR-20a, and BMP2 groups within 2 weeks after the operation, and three maxillary sinus areas in the BMP2+miR-20a group were suppurated. After aspiration + anti-infection treatment, all the rabbits were cured. Furthermore, the symptoms of all the animals improved a lot after additional treatment with gentamicin.

### miR-20a increases the bone density of rabbits with bone defects

**FIGURE 5:**
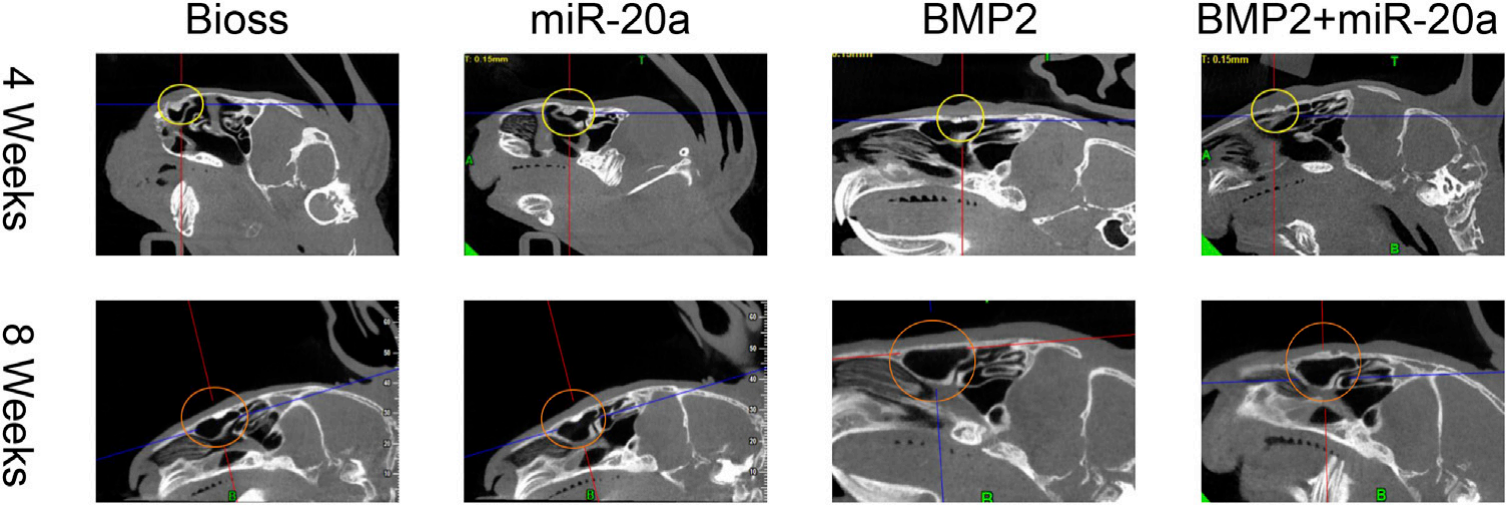
Postoperative CBCT images. Postoperative CBCT images of the skull and maxillofacial bone of rabbits in the Bio-Oss, miR-20a, BMP2, and BMP2+miR-20a groups at 4 and 8 weeks. CBCT, cone beam computed tomography.

Postoperative CBCT images are shown in Figure 5 and the conditions of maxillary sinus elevation of rabbits in each group are shown in Table 1. In the Bio-Oss group, 4 weeks after surgery, the bone density in the visible lifted area was uneven and discontinuous, compared with the surrounding bone; at 8 weeks, the visible lifted area showed clear boundaries, hyper-density shadows, and more continuous edges. In the miR-20a group, at 4 weeks, the visible lifted area exhibited uneven bone density and the bone repair area presented a lot of scattering; at 8 weeks, the boundary between the visible lifted area and the surrounding bone was slightly blurred and a uniform high-density shadow could be observed. In the BMP2 group, most of the visible lifted areas focused on high-density and not continuous bone boundaries at 4 weeks; at 8 weeks, the line dividing the lifted area and the surrounding bone was almost invisible. In the BMP2+miR-20a group, at 4 weeks, irregular high-density and low-density shadows were present in the bone repair area; at 8 weeks, the density of the lifted area was relatively uniform and similar to the density of surrounding bone.

**TABLE 1:** Maxillary sinus elevation of rabbits in each group.

Group	Material	Time after operation		Maxillary sinus number
		4 weeks	8 weeks	
A	Bio-Oss	3	3	6
B	miR-20a	3	3	6
C	BMP2	3	3	6
D	miR-20a + BMP2	3	3	6

BMP2, bone morphogenetic protein 2.

Next, the grayscale values of the deepest, middle, and high levels of the maxillary sinus lift surgery area on CBCT were measured using NNT Viewer software. The results (Table 2) showed that the grayscale values of the deepest (bottom) level of the four groups were significant at 4 weeks after the bone repair operation (*p* < 0.05). At 4 weeks, the differences between the middle and surface (top) grayscale values of the four groups were not statistically significant (all, *p* > 0.05). At 8 weeks, the middle grayscale values of the maxillary sinus lift area of the four groups were significantly different (*p* < 0.05). The deepest (bottom) and high (top)-level grayscale values of the four groups were not different (all, *p* > 0.05). Taken together, miR-20a could increase the bone density of rabbits with bone defects.

**TABLE 2:** Comparison of gray values at the base of maxillary sinus elevation in rabbits.

Position	Group	Number	4 weeks	8 weeks	t	*P*
	Bio-Oss	15	657.03 ± 229.80	875.07 ± 117.17	−3.898	**0.001**
Bottom	miR-20a	15	852.4 ± 199.42	889.37 ± 227.53	0.473	0.64
	BMP2	15	912.8 ± 316.94	860.53 ± 255.78	0.497	0.623
	miR-20a + BMP2	15	991.57 ± 190.14	1030.77 ± 291.68	2.02	0.055
			F = 5.339	F = 1.724		
			***p* = 0.003**	*p* = 0.173		
	Bio-Oss	15	961.7 ± 114.57	966.83 ± 231.71	−2.492	**0.02**
Middle	miR-20a	15	1173.5 ± 350.83	1250.27 ± 348.34	−0.601	0.552
	BMP2	15	965.17 ± 320.57	1080.4 ± 205.08	−1.173	0.251
	miR-20a + BMP2	15	1021.07 ± 287.99	1373.13 ± 465.23	−0.077	0.939
			F = 1.835	F = 4.474		
			*p* = 0.151	***p* = 0.007**		
	Bio-Oss	15	892.13 ± 282.08	912.27 ± 283.15	−2.429	**0.022**
Top	miR-20a	15	832.53 ± 256.71	969.67 ± 367.76	−1.184	0.246
	BMP2	15	748.1 ± 206.91	1001.07 ± 149.67	−3.837	**0.001**
	miR-20a + BMP2	15	739.7 ± 176.59	1166.63 ± 334.71	−2.003	0.057
			F = 1.443	F = 2.045		
			*p* = 0.240	*p* = 0.118		

BMP2*,* bone morphogenetic protein 2.

### miR-20a increases the number of osteoblasts and promotes repair process of the rabbits with bone defects

**FIGURE 6:**
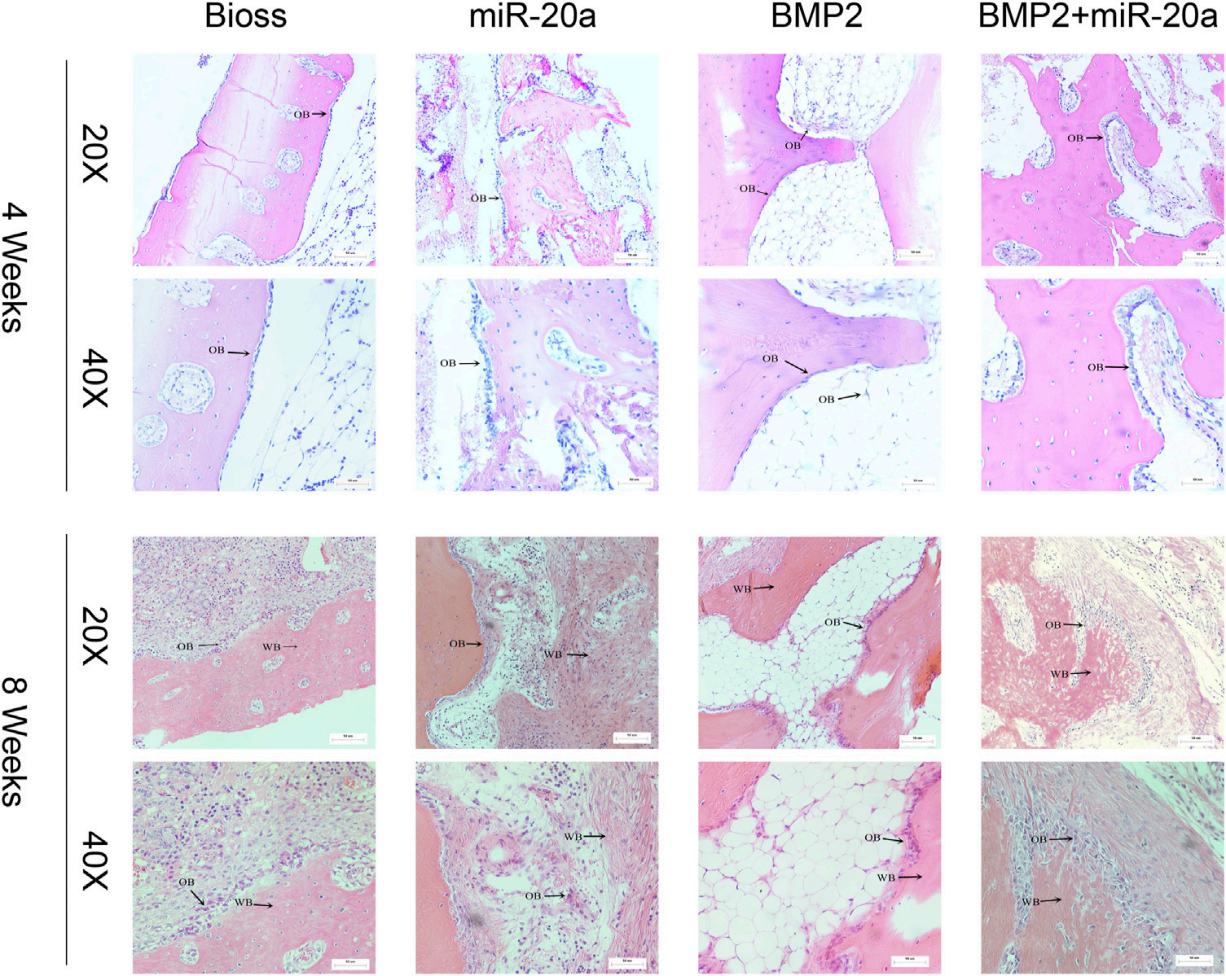
Hematoxylin–eosin staining. Hematoxylin–eosin staining of the bone tissue of rabbits in the Bio-Oss, miR-20a, BMP2, and BMP2+miR-20a groups at 4 and 8 weeks after surgery. OB, osteoblasts; BMP2, bone morphogenetic protein 2.

Images of HE staining are shown in Figure 6. At 4 weeks after surgery, bone tissue surfaces in the Bio-Oss group were covered with monolayer osteoblasts and the cells were loosely networked. In the miR-20a group, bone tissue was covered with multiple layers of osteoblasts. In the BMP2 group, a monolayer of osteoblasts covered the bone tissue surfaces. As for the BMP2+miR-20a group, bone tissue surfaces were covered with a multilayer of osteoblasts and nuclear irregularities could be observed.

At 8 weeks after surgery, the Bio-Oss group exhibited increased woven bones and a large number of bone cells; there were multiple layers of osteoblasts arranged on the surface of the woven bone. The miR-20a group showed a large number of clumps and other polymorphic woven bones; osteoblasts were surrounded by distinct bundles of thicker fibrous connective tissue. The BMP2 group displayed a large number of woven bones with an irregular internal arrangement; there was visible bundle-like, braided tissue and increased osteoblasts on the surface of the woven bone. The BMP2+miR-20a group presented the largest amount of woven bone, with a large number of osteoblasts around the bone tissue.

### miR-20a increases the secretion of collagen type 1 in rabbits with bone defects

**FIGURE 7:**
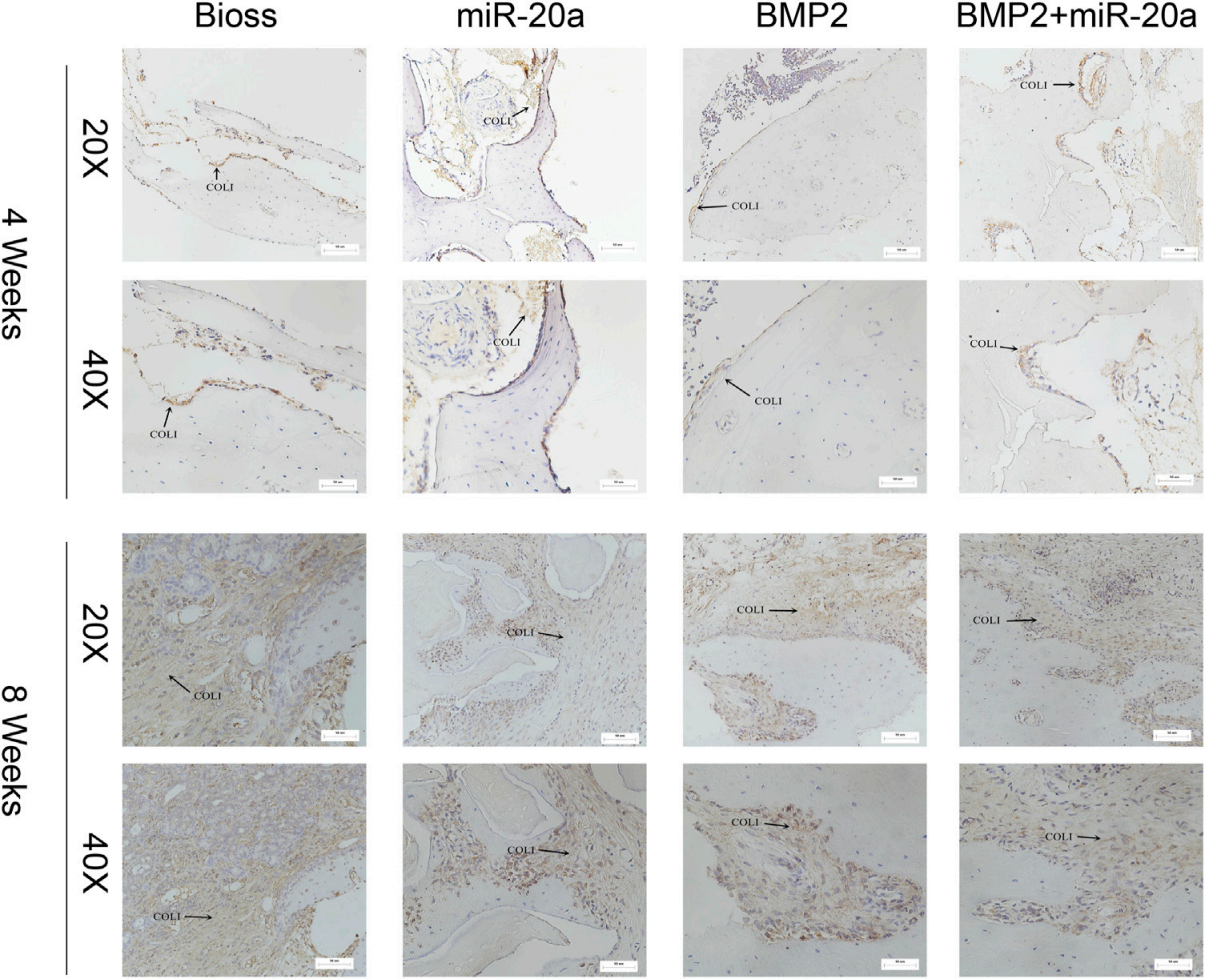
Immunohistochemistry staining for type I collagen. Immunohistochemistry staining for type I collagen of bone tissue of rabbits in the Bio-Oss, miR-20a, BMP2, and BMP2+miR-20a groups at 4 and 8 weeks after surgery.

Immunohistochemistry staining results after surgery are shown in Figure 7. At 4 weeks after surgery, the bone tissue in the BIOS group returned to normal and was covered with a monolayer of osteoblasts, accompanied by a dense distribution of the surrounding type I collagen (COL-1). In the miR-20a group, a large amount of type I collagen was distributed around osteoblasts, with a large depth of staining range and interconnectedness. In the BMP2 group, there was less type I collagen distributed around osteoblasts and less staining could be observed. In the BMP2+miR-20a group, staining was deeper on the surface of normal bone tissue. At 8 weeks after surgery, deep staining covered a large area of bone tissue in the Bio-Oss group, closely surrounding the osteoblasts. In the miR-20a group, deeper staining was observed and the collagen fibers were thinner and tightly connected to each other. In the BMP2 group, deep staining was observed near the woven bone and collagen fiber bundles were thicker and denser. In the BMP2+miR-20a group, there was deep staining of fiber bundles that were thicker and arranged irregularly.

## Discussion

As a miR-17-92 cluster member, miR-20a is expressed in mice and humans and is highly expressed in osteoblasts and bone tissue (Zhou et al., 2014). Our previous studies demonstrated that FSS not only induced the osteogenic differentiation of BMSCs in mice but also promoted the endogenous expression of miR-20a in a time-dependent manner. It is reported that miR-20a downregulates BAMBI and Smad6, promotes the BMP2 signaling pathway, and strengthens FSS-induced osteogenic differentiation in BMSCs. In this study, overexpression of miR-20a promoted the differentiation of BMSCs, enhanced the ALP activity, and facilitated matrix mineralization, whereas anti-miR-20a played a suppressive role in the aforementioned process. Furthermore, high expression of miR-20a activated the BMP2/RUNX2 signaling pathway and increased the mRNA levels and protein levels of BMP2, RUNX2, and SP, whereas they were suppressed by anti-miR-20a. These findings indicated that transcription and translation of BMP2, RUNX2, and SP7 are mediated by miR-20a. In other words, upregulation of miR-20a enhances OS-induced BMSC differentiation *via* the BMP2/RUNX2 signaling pathway.

miR-20a also plays a crucial role in the osteogenic differentiation of human mesenchymal stem cells *via* the BMP2/RUNX2 signaling pathway by targeting BAMBI, Crim1, and PPARγ (Zhang et al., 2011). Moreover, upregulation of miR-20a can transform stem cells derived from human adipose tissue into osteoblasts (Luo et al., 2020). Wang H. demonstrated that miR-20a inhibited the proliferation and osteogenic differentiation of THP-1 cells by downregulating PPARγ (Wang and Shen, 2019). Overall, miR-20a plays an important role in the positive regulation of osteoblastic differentiation. However, it is not clear whether miR-20a can promote bone defect repair in an animal model. Therefore, we constructed a bone defect model of the rabbit maxillary sinus, transfected bone, and mucosa of the maxillary sinus with AAV carrying miR-20a and observed the effect of AAV loaded with miR-20a and BMP2 combined with Bio-Oss bone powder on bone defect repair in this study.

The results of this study show that compared with routine Bio-Oss bone powder treatment, AAV loaded with miR-20a can accelerate bone formation after maxillary sinus lift surgery. This study proposed and confirmed the fact that miR-20a/AAV is able to transfect bone cells and functions similar to BMP2 in repairing rabbit maxillary sinus bone defects; notably, inflammatory cell aggregation has not been proved by any evidence yet. Related studies have verified that miRNA-21 plays a role in osteogenesis by implanting a miRNA-21-modified BMSCs/β-tricalcium phosphate (β-TCP) composite into critical-sized defects (Yang et al., 2019). The animal experiment of Sun et al. (2015) confirmed that overexpression of miRNA-21 promoted osteogenesis and accelerated fracture healing by up-regulating the expression of osteopontin and ALK in BMSCs. Sun et al. (2019) studied miR-26a in a rat model of bone non-union. The results of the radiograph, HE staining, and Masson staining all showed that miR-26a significantly enhanced osteogenesis. In other words, miR-26a promoted fracture healing through the Wnt/β-Catenin signal transduction pathway. Moreover, by inhibiting the expression of sclerostin domain-containing 1 (SOSTDC1), miR-26a induced osteogenic differentiation of MSCs and then promoted the healing of bone nonunion. Shi et al. (2018) transplanted BMSCs overexpressing miR-218 into a mouse model of femoral fracture. Through radiographic and micro-CT examination, mechanical testing, and tissue staining, they found that miR-218 accelerated fracture healing. Ge et al. (2018) claimed that the expression of miR-374b in mice with early fracture was significantly increased. Subsequent *in vitro* cell experiments showed that apart from upregulation of ALP activity in MSCs, miR-374b also significantly increased the expression of osteogenesis-related genes and proteins. However, the opposite results were observed with knockdown of miR-374b. Further study revealed that miR-374b promoted the osteogenic differentiation of MSCs by degrading PTEN, thereby promoting fracture healing. Yuan et al. (2019) used biphasic calcium phosphate scaffold adipose in combination with stem cells transfected with anti-miR-26a-5p for the treatment of rat femoral defects. After treatment, they found improved bone density and an increased number of bone trabeculae in the 8th week. Zhang et al. (2016) synthesized a polylactic acid microsphere-encapsulated miR-26a, and these microspheres were linked to an acellular nanofiber polymer scaffold to control the release of miR-26a, thereby continuously activating endogenous stem cells to repair bone defects.

Our experimental results showed that BMP2 mixed with Bio-Oss bone powder significantly promoted bone repair of maxillary sinus defects in rabbits, but BMP2 and miR-20a may have no synergistic effect in repairing maxillary sinus bone defects. Notably, more inflammatory cells were observed by HE staining in the BMP2 group. Because rhBMP2 promotes bone formation by inducing bone-derived cells into osteoblasts, it can be applied in maxillary sinus elevation. Torrecillas-Martinez et al. (2013) confirmed the osteogenic and osteoinductive capacity of rhBMP2 and supported it as a substitute for autologous bone in maxillary sinus floor elevation. In addition, a research study showed that compared with applying autologous bone graft alone, the final bone density was higher after applying recombinant human BMP2 (rhBMP2) in maxillary sinus floor elevation (Susin et al., 2018). However, the results of other studies have not provided clear evidence regarding the effectiveness of rhBMP2 in maxillary sinus elevation. Briefly, compared with Bio-Oss bone powder, low-dose rhBMP2 and HA can promote bone formation more effectively in the early stage of maxillary sinus elevation (Kim et al., 2015a). Another research showed that the effect of rhBMP2 is similar to that of Bio-Oss bone powder in promoting bone formation when it is used in maxillary sinus elevation surgery (Kim et al., 2015b). Kao et al. utilized a collagen sponge to deliver rhBMP2 in combination with Bio-Oss for maxillary sinus elevation, and they found that the effect of combination treatment on bone regeneration was not as good as applying Bio-Oss alone (Kao et al., 2012). Chun et al. (2020) stated that BMP2 tends to induce many adverse reactions, such as inflammation and bone resorption. After BMP2 treatment, the expression of IL-6 was increased 18-fold in gingival fibroblasts in comparison with the control group. BMP2 also enhanced the inflammatory response of fibroblasts at the time of enhancing osteogenesis differentiation. In our experiment, the BMP2 solution was injected into the bone defect area and inevitably touched the surrounding mucosa, which may enhance the inflammatory response of the surrounding soft tissue and then affect early bone formation.

Chang et al. (2018) pointed out that the combination of BMP or other osteogenic cytokines with scaffold materials can induce new bone formation; however, these exogenous cytokines are prone to rapid degradation and have a short half-life *in vivo,* so they fail to play a continuous regulatory role. Fortunately, miRNA can endogenously promote the self-secretion signal of bone formation, produce osteoblast-related cytokines, and achieve the healing of large bone defects. In other words, the application of miRNA combined with scaffold materials can anchor specific tissues faster to achieve local tissue delivery therapy. In the future, further research on the construction of scaffold materials is required to more precisely control the release of miRNA, thereby providing a new therapeutic regimen for bone defects.

## Conclusion

To sum up, upregulation of miR-20a promotes OS-induced osteogenic differentiation by activating the BMP2/RUNX2 signaling pathway. In addition, miR-20a/AAV mixed with Bio-Oss bone powder significantly facilitates the bone repair of maxillary sinus defects in rabbits. However, it is worth noting that miR-20a and BMP2 may have no synergistic effect. More experiments need to be performed to elucidate the relationship between miR-20a and BMP2.

## Data Availability

The original contributions presented in the study are included in the article/Supplementary Materials; further inquiries can be directed to the corresponding author.

## References

[B1] Bathla S. C. Fry R. R. Majumdar K. (2018). Maxillary sinus augmentation. J. Indian Soc. Periodontol. 22 (6), 468–473. 10.4103/jisp.jisp_236_18 30631223 PMC6305100

[B2] Cha H. S. Kim J. W. Hwang J. H. Kang M. A. (2016). Frequency of bone graft in implant surgery. Maxillofac. Plast. Reconstr. Surg. 38 (1), 19. 10.1186/s40902-016-0064-2 27077072 PMC4819798

[B3] Chang C. C. Venø M. T. Chen L. Ditzel N. Le D. Q. S. Dillschneider P. (2018). Global MicroRNA profiling in human bone marrow skeletal-stromal or mesenchymal-stem cells identified candidates for bone regeneration. Mol. Ther. 26 (2), 593–605. 10.1016/j.ymthe.2017.11.018 29331291 PMC5835027

[B4] Chun J. Jung J. Lee J. Oh S. H. Kwon Y. D. (2020). Osteogenic differentiation and inflammatory response of recombinant human bone morphogenetic protein-2 in human maxillary sinus membrane-derived cells. Exp. Ther. Med. 20 (5), 81. 10.3892/etm.2020.9208 32968438 PMC7500044

[B5] Danesh-Sani S. A. Loomer P. M. Wallace S. S. (2016). A comprehensive clinical review of maxillary sinus floor elevation: Anatomy, techniques, biomaterials and complications. Br. J. Oral Maxillofac. Surg. 54 (7), 724–730. 10.1016/j.bjoms.2016.05.008 27235382

[B6] Ge J. B. Lin J. T. Hong H. Y. Sun Y. J. Li Y. Zhang C. M. (2018). MiR-374b promotes osteogenic differentiation of MSCs by degrading PTEN and promoting fracture healing. Eur. Rev. Med. Pharmacol. Sci. 22 (11), 3303–3310. 10.26355/eurrev_201806_15149 29917179

[B7] Guo T. Q. Gulati K. Shen Z. Y. Han P. P. Fan Z. (2020). Therapeutic outcomes of non-grafted and platelet concentrations-grafted transcrestal maxillary sinus elevation (tsfe): A systematic review and meta-analysis. Sci. Rep. 10 (1), 5935. 10.1038/s41598-020-62407-y 32245996 PMC7125188

[B8] Kao D. W. Kubota A. Nevins M. Fiorellini J. P. (2012). The negative effect of combining rhBMP-2 and Bio-Oss on bone formation for maxillary sinus augmentation. Int. J. Periodontics Restor. Dent. 32 (1), 61–67. 22254226

[B9] Kim H. J. Chung J. H. Shin S. Y. Shin S. I. Kye S. B. Kim N. K. (2015a). Efficacy of rhBMP-2/hydroxyapatite on sinus floor augmentation: A multicenter, randomized controlled clinical trial. J. Dent. Res. 94 (9), 158S–65S. 10.1177/0022034515594573 26185033

[B10] Kim H. J. Lee J. S. Shin H. K. Kim J. S. Yun J. H. Cho K. S. (2015b). Prospective randomized, controlled trial of sinus grafting using Escherichia-coli-produced rhBMP-2 with a biphasic calcium phosphate carrier compared to deproteinized bovine bone. Clin. Oral Implants Res. 26(12), 1361–1368. 10.1111/clr.12471 25186180

[B11] Kong X. H. Shi S. F. Hu H. J. Wang J. X. (2021). MicroRNA-20a suppresses RANKL-modulated osteoclastogenesis and prevents bone erosion in mice with rheumatoid arthritis through the TLR4/p38 pathway. J. Biol. Regul. Homeost. Agents 35 (3), 921–931. 10.23812/20-604-A 34212684

[B12] Lee J. Susin C. Rodriguez N. A. de Stefano J. Prasad H. S. Buxton A. N. (2013). Sinus augmentation using rhbmp-2/ACS in a mini-pig model: Relative efficacy of autogenous fresh particulate iliac bone grafts. Clin. Oral Implants Res. 24 (5), 497–504. 10.1111/j.1600-0501.2011.02419.x 22276816

[B13] Li Y. Zhang Z. J. Gu X. G. Jin Y. Feng C. Yang S. Y. (2020). MicroRNA-21 affects mechanical force-induced midpalatal suture remodelling. Cell Prolif. 53 (1), e12697. 10.1111/cpr.12697 31713930 PMC6985676

[B14] Luo T. Yang X. Sun Y. Huang X. Zou L. Liu J. (2020). Effect of MicroRNA-20a on osteogenic differentiation of human adipose tissue-derived stem cells. Cells Tissues Organs 208 (3-4), 148–157. 10.1159/000506304 32097913

[B15] Mai Z. Xiao F. Liu G. Wang Y. Xie S. Ai H. (2022). MiR-20a: A mechanosensitive microRNA that regulates fluid shear stress-mediated osteogenic differentiation via the BMP2 signaling pathway by targeting BAMBI and SMAD6. Ann. Transl. Med. 10 (12), 683. 10.21037/atm-22-2753 35845505 PMC9279817

[B16] Mai Z. H. Peng Z. L. Zhang J. L. Chen L. Liang H. Y. Cai B. (2013). miRNA expression profile during fluid shear stress-induced osteogenic differentiation in MC3T3-E1 cells file during fluid shear stress-induced osteogenic differentiation in MC3T3-E1 cells. Chin. Med. J. (Engl.) 126, 1544–1550. 23595392

[B17] Meng Y. Huang X. Wu M. Yang X. Q. Liu Y. (2020). The effect of autologous platelet concentrates on maxillary sinus augmentation: A meta-analysis of randomized controlled trials and systematic review. Biomed. Res. Int. 2020, 7589072. 10.1155/2020/7589072 32626762 PMC7315322

[B18] Shi L. Feng L. Liu Y. Duan J. Q. Lin W. P. Zhang J. F. (2018). MicroRNA-218 promotes osteogenic differentiation of mesenchymal stem cells and accelerates bone fracture healing. Calcif. Tissue Int. 103 (2), 227–236. 10.1007/s00223-018-0410-8 29523928

[B19] Sun L. Li Z. Xue H. Ma T. Ren C. Li M. (2019). MiR-26a promotes fracture healing of nonunion rats possibly by targeting SOSTDC1 and further activating Wnt/β-catenin signaling pathway. Mol. Cell Biochem. 460 (1-2), 165–173. 10.1007/s11010-019-03578-9 31313025

[B20] Sun Y. Xu L. Huang S. Hou Y. Liu Y. Chan K. M. (2015). mir-21 overexpressing mesenchymal stem cells accelerate fracture healing in a rat closed femur fracture model. Biomed. Res. Int. 2015, 1–9. 10.1155/2015/412327 PMC4386680 25879024

[B21] Susin C. Lee J. Fiorini T. Moreno de Freitas R. Chiu H. C. Prasad H. S. (2018). Sinus augmentation using rhBMP-2/ACS in a mini-pig model: Influence of an adjunctive ceramic bone biomaterial. J. Clin. Periodontol. 45(8), 1005–1013. 10.1111/jcpe.12921 29757470

[B22] Torrecillas-Martinez L. Monje A. Pikos M. A. Ortega-Oller I. Suarez F. Galindo-Moreno P. (2013). Effect of rhbmp-2 upon maxillary sinus augmentation: A comprehensive review[J]. Implant Dent. 22 (3), 232–237. 10.1097/ID.0b013e31829262a8 23648577

[B23] Wang H. Hu Z. B. Shi F. Dong J. J. Dang L. Wang Y. X. (2018). Osteoblast-targeted delivery of miR-33-5p attenuates osteopenia development induced by mechanical unloading in mice. Cell Death Dis. 9, 170. 10.1038/s41419-017-0210-5 29415986 PMC5833703

[B24] Wang H. Shen Y. (2019). MicroRNA-20a negatively regulates the growth and osteoclastogenesis of THP-1 cells by downregulating PPARγ. Mol. Med. Rep. 20 (5), 4271–4276. 10.3892/mmr.2019.10676 31545439

[B25] Xiao C. Pan X. F. Zhang B. Huang W. Pei F. Luo T. (2021). miR-20a-5p contributes to osteogenic differentiation of human dental pulp stem cells by regulating BAMBI and activating the phosphorylation of Smad5 and p38. Stem Cell Res. 12 (1), 421. 10.1186/s13287-021-02501-8 PMC8296686 34294156

[B26] Yang C. Liu X. Zhao K. Zhu Y. Hu B. Zhou Y. (2019). miRNA-21 promotes osteogenesis via the PTEN/PI3K/Akt/HIF-1α pathway and enhances bone regeneration in critical size defects. Stem Cell Res. Ther. 10 (1), 65. 10.1186/s13287-019-1168-2 30795815 PMC6387542

[B27] Yuan X. Y. Han L. Lin H. Guo Z. Y. Huang Y. L. Li S. S. (2019). The role of antimiR-26a-5p/biphasic calcium phosphate in repairing rat femoral defects. Int. J. Mol. Med. 44 (3), 857–870. 10.3892/ijmm.2019.4249 31257525 PMC6658005

[B28] Zhang J. F. Fu W. M. He M. L. Xie W. D. Lv Q. Wan G. (2011). MiRNA-20a promotes osteogenic differentiation of human mesenchymal stem cells by co-regulating BMP signaling. RNA Biol. 8, 829–838. 10.4161/rna.8.5.16043 21743293

[B29] Zhang X. Li Y. Chen Y. E. Chen J. Ma P. X. (2016). Cell-free 3D scaffold with two-stage delivery of miRNA-26a to regenerate critical-sized bone defects. Nat. Commun. 7, 10376. 10.1038/ncomms10376 26765931 PMC4735608

[B30] Zhou M. Ma J. Chen S. (2014). MicroRNA-17-92 cluster regulates osteoblast proliferation and differentiation. Endocrine 45, 302–310. 10.1007/s12020-013-9986-y 23673870

